# An examination of the relationship between shame, guilt and self-harm: A systematic review and meta-analysis

**DOI:** 10.1016/j.cpr.2019.101779

**Published:** 2019-11

**Authors:** Kate Sheehy, Amna Noureen, Ayesha Khaliq, Katie Dhingra, Nusrat Husain, Eleanor E. Pontin, Rosanne Cawley, Peter J. Taylor

**Affiliations:** a Institute of Psychology, Health and Society, University of Liverpool, United Kingdom; b Pakistan Institute of Living & Learning, Karachi, Pakistan; c School of Social Sciences, Leeds Beckett University, LS1 3HE England, United Kingdom; d Division of Psychology & Mental Health, School of Health Sciences, University of Manchester, Manchester Academic Health Sciences Centre, Manchester, United Kingdom

**Keywords:** Shame, Guilt, Self-harm, Non-suicidal self-injury (NSSI), Suicide, Systematic review

## Abstract

Self-harm is a major public health concern associated with suicide risk and significant psychological distress. Theories suggest that aversive emotional states are an important process that drives self-harm. Shame and guilt may, in particular, be important emotions in self-harm. This review therefore sought to provide a systematic review and meta-analysis of the relationship between shame, guilt, and self-harm. A systematic search of electronic databases (PsycINFO; Medline; CINAHL Plus; Web of Science and ProQuest) was undertaken to identify studies measuring shame, guilt and self-harm (including suicidal and non-suicidal behaviour). Meta-analysis was undertaken where papers focused on the same subtype of shame or guilt and shared a common outcome. Thirty studies were identified for inclusion. Most forms of shame were associated with non-suicidal self-injury (NSSI), but research was sparse concerning suicidal behaviour. Fewer studies examined guilt and findings were more varied. Methodological issues included a paucity of longitudinal designs and lack of justification for sample sizes. Results of this review support the link between shame and self-harm, particularly NSSI. The direction of this relationship is yet to be established. Clinically, consideration should be given to the role of shame amongst individuals who present with NSSI. This review was pre-registered on PROSPERO (CRD42017056165).

## Highlights

• Shame has a positive association with self-harm.
• Guilt proneness does not appear to be related to self-harm.
• However, exposure to state guilt is greater in those with a history of self-harm.
• Shame should be considered in psychological assessments with those who self-harm.
• Longitudinal studies in this area are lacking.

## 1 Introduction

Self-harm poses a significant public health concern worldwide, because of its high prevalence (Muehlenkamp, Claes, Havertape, & Plener, 2012; Swannell, Martin, Page, Hasking, & St John, 2014), and association with subsequent suicide risk (Hawton et al., 2015; Ribeiro et al., 2016). It is also often indicative of psychological distress and additional need (Goldman-Mellor et al., 2014), and reduced life expectancy from any cause (approximately 26 years of life lost; Bergen et al., 2012). Self-harm refers to the deliberate destruction or damage to one's own body tissue, irrespective of suicidal intent, and can be applied to a range of behaviours including overdose, cutting, burning, and self-battery (National Collaborating Centre for Mental Health, 2004; Royal College of Psychiatrists, 2010). Self-harm therefore encompasses suicidal behaviour (i.e. suicide attempts) and non-suicidal self-injury (NSSI; Klonsky, 2011), as well as behaviours where the level of suicidal intent is unclear or ambiguous. Recent reviews suggest that one of the most commonly reported reasons for self-harm is around coping with or regulating difficult emotional states (Edmondson, Brennan, & House, 2016; Taylor et al., 2018). For example, this function is endorsed by 71% (95% CI: 63–78%) of those who engage in NSSI (Taylor, Jomar, et al., 2018). Thus, mechanisms involving exposure to and regulation of emotional states appear key to understanding self-harm (Nock, 2009). A better understanding of these mechanisms can help inform the development and adaptation of interventions for those who struggle with self-harm (Muehlenkamp, 2006). Certain emotions appear especially important in understanding self-harm (Klonsky, 2009). The current review focuses on two such emotions, shame and guilt.

Shame and guilt have been described as self-conscious, ‘moral’ emotions, which arise in response to an evaluation of the self (Tangney & Dearing, 2002; Tangney, Stuewig, & Mashek, 2007). Although routinely considered in tandem, shame and guilt are thought to represent distinct, yet overlapping, emotional experiences (Tangney et al., 2007). Current thinking regarding this distinction points to a differential focus on the self, versus one's behaviour. At its core, shame can be seen as a cognitive affective construct, comprising negative judgements of the self (Chou et al., 2018). These judgements are global, undesirable, and characterised by an evaluation of the self as inherently flawed, inadequate or bad (Blythin et al., 2018; Carden, Saini, Seddon, Watkins, & James Taylor, 2018; Gilbert & Procter, 2006). By contrast, guilt is concerned with one's behaviour, and the negative evaluation of this (Tangney et al., 2007; Tangney & Dearing, 2002). Hence, the object of focus is something done by the individual that is perceived as bad or wrong, rather than the individual themselves. As a result, the phenomenological experiences of guilt and shame are said to diverge significantly (Lewis, 1971).

Traditionally, the conceptualisation of shame has centred upon the individual's perception of themselves. However, some researchers have distinguished between this and an individual's representation of how they are perceived by others (specifically the individual's perception of being negatively judged by others), referred to as ‘external’ shame (Gilbert, 1997, Gilbert, 1998). In addition, shame may be thought to arise in relation to different aspects of the self, such as one's character, behaviour, or body (Andrews, Qian, & Valentine, 2002). As a result, a range of psychometric measures have been developed and used to assess these various components of shame. No such distinctions have been made in relation to guilt as far as we are aware. Furthermore, whilst it is acknowledged that shame and guilt may occur in relation to specific incidents or events, it is now also recognised that some individuals have a greater tendency, or proneness, to experience feelings of shame or guilt across a range of situations (Tangney, 1990).

Both shame and guilt may be experienced as unwanted or aversive emotional states. However, literature suggests that shame may be particularly pernicious due to its close ties with an individual's sense of self (Lewis, 1971). Indeed, shame is closely linked with various psychological difficulties including depression, psychosis, Post-Traumatic Stress Disorder (PTSD), and eating disorders (Blythin et al., 2018; Carden et al., 2018; Kim, Thibodeau, & Jorgensen, 2011; Pugh, Taylor, & Berry, 2015). Across the available research, there is evidence that shame is more robustly associated with psychological difficulties and that when adjusting for overlapping shame, guilt at times is no longer associated with mental health difficulties (Blythin et al., 2018; Kim et al., 2011; Pugh et al., 2015). Nonetheless, guilt too may be experienced as painful, and may give rise to feelings of regret or remorse (Pugh et al., 2015; Tangney, Miller, Flicker, & Barlow, 1996). Whilst guilt may lead an individual to engage in reparative action to address perceived problematic behaviour (Tangney & Dearing, 2002), responses to shame are typically less adaptive and include rumination (Cheung, Gilbert, & Iron, 2004), submission (Gilbert, Pehl, & Allan, 1994), avoidance (Schoenleber & Berenbaum, 2012), and attempts to conceal oneself or one's perceived faults (Tangney et al., 1996). In light of the available literature, it is hypothesised that shame will show a stronger relationship with self-harm than guilt.

Theoretical models have typically focused either on suicidal behaviour or NSSI, rather than the broader construct of self-harm. Theoretical models of NSSI explicitly suggest that NSSI is maintained by negative reinforcement, characterised by escape from unpleasant internal states, including emotions like shame and guilt (Chapman, Gratz, & Brown, 2006; Hasking, Whitlock, Voon, & Rose, 2017; Nock, 2009). Some theorists have developed these ideas further by implying a specific role of shame or guilt in the aetiology and maintenance of NSSI. For example, some NSSI may arise out of beliefs about the self as deserving of punishment (Glassman, Weierich, Hooley, Deliberto, & Nock, 2007; Nock, 2009), which could be a consequence of strong feelings of shame. Schoenleber and Berenbaum (2012), for example, propose that individuals may engage in NSSI as a means of managing feelings of shame. Theories of suicidal behaviour have also posited that strong negative emotions may drive suicidal behaviour (Baumeister, 1990; Williams, 1997). The Interpersonal Theory of suicide (Joiner, 2005; Van Orden et al., 2010) suggests feelings of burdensomeness are key to the desire for suicide, and that feelings of self-hate are a facet of this construct (Van Orden et al., 2010; Van Orden, Cukrowicz, Witte, & Joiner, 2012). Since shame is the emotion perhaps most synonymous with self-hate it may therefore be relevant in driving suicidal urges. Shame may be part of the mechanism explaining the increased risk of self-harm in some marginalized groups, such as those who are lesbian, gay or bisexual (Taylor, Dhingra, Dickson, & McDermott, 2018), or those belonging to alternative subcultures (Hughes, Knowles, Dhingra, Nicholson, & Taylor, 2018). For example, experiences of rejection associated with belonging to a marginalized group (e.g. being lesbian, gay, bisexual or transgender) are associated with self-harm risk (Cawley, Pontin, Touhey, Sheehy, & Taylor, 2019).

The current research aims to provide a systematic review and meta-analysis of the available literature pertaining to self-harm and its relationships with shame and guilt. We will appraise the weight of evidence concerning the relationship between these constructs, and via the meta-analysis quantify the size of these associations. It has been noted that shame and guilt overlap with one another, and also with depressive symptoms. In addition to focussing on bivariate associations, we also review associations whilst adjusting for guilt (when the effect involves shame) or shame (when the effect involves guilt) and depression.

## 2 Method

### 2.1 Protocol registration

A systematic review protocol was developed and pre-registered online with PROSPERO (CRD42017056165). This review followed the PRISMA reporting guidelines (Moher, Liberati, Tetzlaff, Altman, & The, 2009). Departures from protocol include the addition of meta-analyses, which were included to provide a further summary of associations, the exclusion of ETHOS as a database (as it is limited to UK dissertations), the expansion of the review team, and the secondary outcome concerning help-seeking being dropped in light of recent existing reviews in this area (Rowe et al., 2014).

### 2.2 Search strategy

First, scoping searches were undertaken to aid the identification of relevant search terms. Following this, four databases were searched (PsycINFO; Medline; CINAHL Plus; Web of Science) to identify relevant published studies (from earliest records until March 2017). These searches were later updated to December 2018. The following search terms were used related to: (a) self-harm: *NSSI* OR suicid* OR *self-harm* OR self-injur* OR *self-mutilation* OR *overdose* OR *DSH* OR parasuicid*; and (b) shame or guilt: *ashamed* OR shame* OR guilt* OR *self-blame* OR *self-disgust*. Search terms for the two groups were combined using the Boolean operator “AND”. The thesis and dissertation database ProQuest was also searched to identify relevant studies in the grey literature. First, titles and abstracts were screened independently by a single researcher. Following this, the full texts of the remaining articles were read to determine eligibility for inclusion. This was carried out independently by two researchers, with discrepancies addressed through discussion with a third author. Following the identification of included studies, reference lists of these papers were hand searched and corresponding authors were also emailed to identify any further potentially eligible studies.

### 2.3 Inclusion and exclusion criteria

Studies included in the review were required to meet the following inclusion criteria: i) quantitative research studies, ii) comprising original research, iii) written in English, iv) measuring shame and/or guilt, v) measuring self-harm history or frequency (including NSSI and suicidal behaviour), vi) providing adequate information to estimate associations between variables. Studies using measures that conflated the constructs of shame and guilt were excluded. For example, the Positive and Negative Affect scale has a guilt subscale that has items referring to feeling “ashamed” (Watson & Clark, 1994). Similarly, we excluded measures of shame focused on specific behaviours due to the overlap with guilt. Studies that assessed shame or guilt using a single item measure were also not included in the review, including studies in which guilt was assessed solely as part of a depression or mania measure, such as the Beck Depression Inventory (Beck, Steer, & Brown, 1996), as such measures may not provide a valid and accurate measure of the key constructs. Measures of shame or guilt related to experiences of trauma or grief or consisting of psychotic symptoms (i.e. delusional guilt) were also excluded, since these arguably have a distinct phenomenology relative to shame and guilt more generally.

### 2.4 Risk of bias assessment

Studies included in the review were evaluated for risk of bias using an adapted version of the Agency for Healthcare Research and Quality (AHRQ; Williams, Plassman, Burke, Holsinger, & Benjamin, 2010) risk of bias tool. The AHRQ has previously been adapted for use in systematic reviews of self-injurious behaviour and associated constructs (Hughes et al., 2018; Taylor, Hutton, & Wood, 2015). The tool assesses risk of bias over eleven domains, including the validity of measures used, unbiased selection of participants, and appropriateness of analytic methods. Ratings were made independently by two research team members for each study, and then compared, with discrepancies resolved through discussion. These ratings were then used to identify common risks of bias across the literature as well as areas of strength.

### 2.5 Data extraction

Data extraction was undertaken independently by two research team members for each study, and then compared, with discrepancies resolved through discussion. Extracted data included study details (author, date, study location), study design information (type of design, number of groups, recruitment method), participant characteristics (target sample, age, gender), measures used, and results of analyses.

### 2.6 Meta-analytic calculations

Bivariate associations between shame, guilt and self-harm were grouped according to the emotion type (shame, guilt) and subtype (e.g. bodily shame, external shame, shame proneness) and outcome (NSSI, suicide attempt or self-harm not otherwise specified). Where two or more effects were grouped together these were aggregated via meta-analysis. This approach allowed the effects for different subtypes of shame or guilt to be compared, but it also meant that meta-analyses often had few included studies. We therefore also grouped studies by emotion type (shame or guilt), irrespective of subtype, and outcome. This allowed for effects from a larger set of studies to be combined by including studies investigating different subtypes of shame or guilt together in the same meta-analysis.

A random-effects model was adopted for all meta-analyses to accommodate the expected heterogeneity between studies in terms of sample, design, and measurement. The DerSimonian and Laird (1986) inverse variance estimator, within STATA 14 (StataCorp, 2015) was initially used. Whilst the DL estimator is commonly used, it has been noted that Restricted Maximum Likelihood (REML) estimator may better estimate between-study variance within random effects meta-analysis of continuous outcomes (Veroniki et al., 2014). We therefore repeated all meta-analyses using the REML estimator as well using the METAAN package (Kontopantelis & Reeves, 2010).

Within samples associations were captured with the correlation coefficient, *r*, whilst group differences were captured using cohen's *d*. Where necessary, effects were converted between effect size metrics following the steps outlined by Borenstein, Hedges, Higgins, and Rothstein (2009). For the meta-analyses undertaken on higher-order groupings of studies, a single effect size was taken from each study. Where studies included multiple different scales of the same emotion (e.g. shame) these effects were first combined (following Borenstein et al., 2009). For one study this was not possible due to lack of information about the scales (Rusch et al., 2007) and so the effect size associated with the most commonly used scale (the TOSCA-3) was used. The *I*^*2*^ statistic was used to determine the impact of heterogeneity between studies (Higgins & Thompson, 2002).

## 3 Results

### 3.1 Study characteristics

**Fig. 1:**
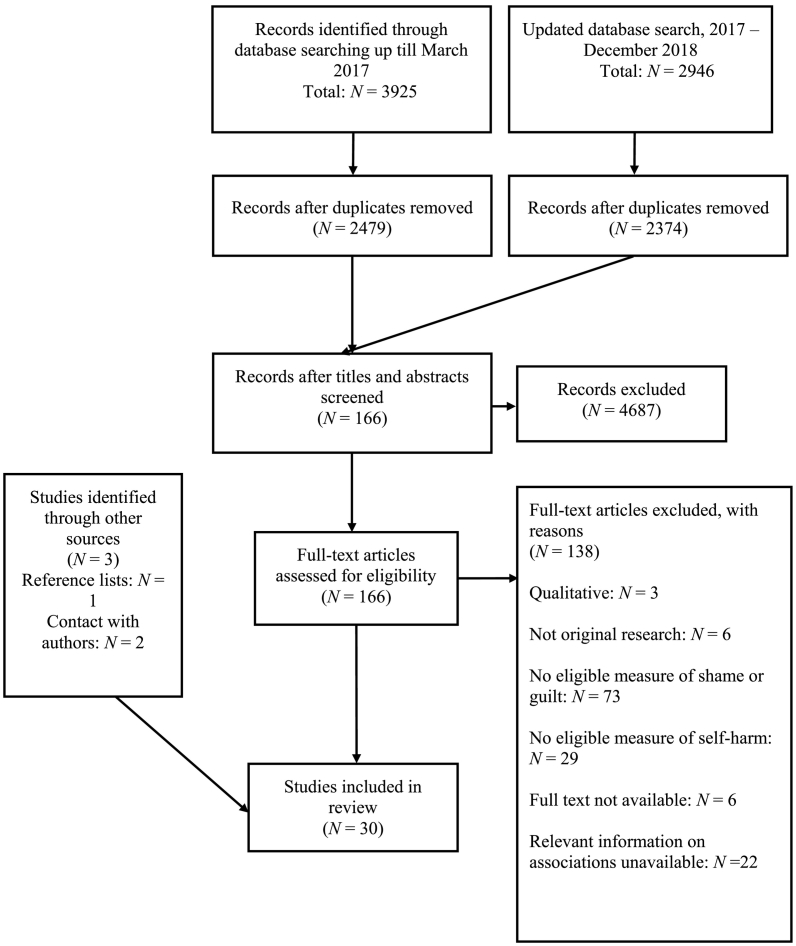
Flow diagram of included studies.

Thirty eligible papers were identified for inclusion in the review. A flow diagram of the screening process from identification through to inclusion is presented in Fig. 1. Study characteristics are summarised in Table 1. All but two studies provided cross-sectional data on the relationship between shame, guilt and self-harm. All studies came from Western countries, most commonly the US (*k* = 18) and UK (*k* = 6). A large number of studies came from MSc or doctoral dissertations (*k* = 14), though the majority derived from peer-reviewed journals (*k* = 16). A single study was unpublished, with data made available by the author. (See Table 2)

**Table 1:** Summary of study characteristics.

Authors, years & country	Design	Participant characteristics	Comparison group characteristics (if applicable)	Self-harm measures	Shame/guilt measures
Brown et al. (2009), US	Longitudinal	N = 77 women seeking psychotherapy for BPD and self-harm; Mean age = 30.0 years, SD = 7.3	–	Suicide Attempt Self-Injury Interview (SASII)	Shame items adapted from the Harder Personal Feelings Questionnaire (PFQ2)
Bryan, Morrow, Etienne, and Ray-Sannerud (2013), US	Cross-sectional	N = 151 outpatients at military mental health clinics; Mean age = 34.12 years, SD = 8.41; Female = 36%	–	Self-Injurious Thoughts and Behavior Interview (SITBI)	The Harder Personal Feelings Questionnaire (PFQ2)
Donhauser (2007), US	Cross-sectional	N = 51 adult childhood sexual abuse survivors; 18–65 years; Female = 84%	–	Deliberate Self-Harm Inventory (DSHI)	Internalised Shame Scale (ISS)
Duggan et al. (2015), Canada	Longitudinal	N = 120 high school students; Mean age = 12.34 years, SD = 0.48; Female = 56%	–	How I Deal with Stress Questionnaire (HIDS)	Objectified Body Consciousness Scale – Youth (OBCS-Y)
Erchull, Liss, and Lichiello (2013); US	Cross-sectional	N = 160 Adult females; Mean age = 23.12, SD = 3.69	–	Deliberate Self-Harm Inventory (DSHI)	Objectified Body Consciousness Scale (OBCS)
Etzel (2004), US	Cross-sectional	N = 65 female victims of intimate partner violence; Mean age = 34.2 years, SD = 8.4	–	Habit Questionnaire for self-injurious behavior	Experience of Shame Scale (ESS)
Fieldman (1989), US	Cross-sectional	N = 30 acute adolescent psychiatric inpatients; Mean age = 15.8 years; Female = 66.7%	–	The Reynolds Suicide Ideation Questionnaire (RSIQ)	Guilt Scale
Gandy (2013), US	Cross-sectional	N = 103 adult survivor of childhood sexual abuse; Mean age = 39.88 years, SD = 13.82; Female = 71.8%	–	modified Suicidal Behavior Questionnaire, (SBQ)	Test of Self-Conscious Affect - 3 (TOSCA-3)
Izadi (2014), US	Cross -sectional	N = 50 individuals with a history of NSSI; Mean age = 27.0 years, SD = 7.1; Female = 74%	–	Deliberate Self-Harm Inventory (DSHI); modified Suicidal Behavior Questionnaire, (SBQ)	Test of Self-Conscious Affect - 3 (TOSCA-3); Shame Variability Questionnaire (SVQ; unpublished)
Kealy, Spidel, and Ogrodniczuk (2017), Canada	Cross -sectional	N = 68 female psychiatric outpatients; Mean age = 36.6 years, SD = 12.0	–	Suicidal Behavior Questionnaire - Revised (SBQ-R)	The Harder Personal Feelings Questionnaire (PFQ2)
Kealy (2019), Canada	Cross -sectional	N = 137 psychiatric outpatients; Mean age = 33.39, SD = 11.98; Female = 69%	–	Item taken from the McLean screening instrument for Borderline Personality Disorders (MSI-BPD)	The Harder Personal Feelings Questionnaire (PFQ2)
Lamb (2004), UK	Cross-sectional	N = 30 psychiatric inpatients and outpatients; Mean age = 37.67 years, SD = 12.4; Female = 86.7%	–	Self-harm Inventory (SHI)	Internalised Shame Scale (ISS)
Lester (1998), US	Cross-sectional	N = 116 university students; Mean age = 21.9 years, SD = 4.6; Female = 67.2%	–	Beck Depression Inventory (BDI)	Shame and Guilt scale
Mallindine (2002), UK	Cross-sectional	N = 21 male inpatients in high secure settings with a NSSI history; Mean age = 31.1 years; SD = 6.99 N = 13 female inpatients in a high secure setting with a NSSI history; Mean age = 28.1; SD = 5.33	N = 15 male inpatients in high secure settings without a history of NSSI; Mean age = 39.8 years; SD = 8.58	Information from clinical records	Other As Shamer scale (OAS)
McLeod (2002), Canada	Cross-sectional	N = 236 psychology university students; Mean age = 19.2 years, SD = 1.0; Female = 72.4%	–	Suicidal Ideation and Behavior Questionnaire (SIBQ)	Test of Self-Conscious Affect (TOSCA); Harder The Harder Personal Feelings Questionnaire (PFQ2); The Domains of Shame Questionnaire (developed within this research)
Milligan and Andrews (2005), UK	Cross-sectional	N = 89 female prisoners; Mean age = 31.8 years, SD = 9.37	–	Impulsive Behavior scale (IBS)	Experience of Shame Scale (ESS)
Nelson and Muehlenkamp (2012), US	Cross-sectional	N = 341 university students; Mean age = 20.2 years, SD = 2.0; Female = 82.4%	–	The Deliberate Self-Harm Inventory (DSHI)	Objectified Body Consciousness Scale (OBCS)
Paulsen (2013), US	Cross-sectional	N = 56 adults survivors of childhood sexual abuse; Mean age = 41.7 years; SD = 15.0; Female = 34%	–	Non Suicidal Self Injury Interview (NSSII)	Test of Self-Conscious Affect - 3 (TOSCA-3)
Pritchard (2009), Online (Canada, UK, US, Australia, New Zealand)	Cross-sectional	N = 54 adults reporting self-harm; Mean age = 21.85 years, SD = 3.58; Female = 66.7% N = 18 adults reporting eating disorders; Mean age = 24.28 years, SD = 5.46; Female = 94.4% N = 106 adults reporting eating disorder and self-harm; Mean age = 23.0 years, SD = 6.65; Female = 90.56%	–	Deliberate Self-Harm Inventory (DSHI)	Objectified Body Consciousness Scale (OBCS)
Rusch et al. (2007), Germany	Cross-sectional	N = 60 female inpatients diagnosed with BPD; Mean age = 27.8 years, SD = 6.9 N = 30 female inpatients with Social Phobia; Mean age = 35.1, SD = 11.9	N = 60 female healthy comparison group; Mean age = 26.6, SD = 7.4	Psychiatric interview	Test of Self-Conscious Affect - 3 (TOSCA-3); The Harder Personal Feelings Questionnaire (PFQ2)
Rutherford (2015), US	Cross-sectional	N = 126 women from mental health clinics and hospitals; Mean age = 34 years, SD = 8.4	–	Study specific measure of suicidal behaviour	Experience of Shame Scale (ESS)
Schaefer (2014), US	Cross-sectional	N = 511 prison inmates; Mean age = 32.19 years, SD = 10.05; Female = 32%	–	Study specific measure of suicidal behaviour	Test of Self-Conscious Affect for Socially Deviant populations (TOSCA-SD)
Schoenleber (2013), US	Cross-sectional	N = 61 female university students; Mean age = 18.9 years, SD = 0.8 N = 54 women living in the community; Mean age = 24.8 years, SD = 6.6	–	Inventory of Statements About Self-Injury (ISAS)	Test of Self-Conscious Affect - 3 (TOSCA-3)
Seidlitz, Conwell, Duberstein, Cox, and Denning (2001), US	Cross-sectional	N = 47 inpatients aged over 50 years with depression and a history of suicide attempts; Mean age = 59.5 years, SD = 8.3; Female = 60%	N = 38 inpatients aged over 50 years with depression and no history of suicide attempts; Mean age = 62.9 years, SD = 10.3; Female = 55%	Study specific measure of suicidal behaviour	Emotional traits assessed via NEO Personality Inventory - Revised (NEO-PI-R)
Taylor, Jomar, et al. (2018), UK	Cross-sectional	N = 51 adults who report having engaged in NSSI in the past year; 96% aged under 30 years; Female = 80% N = 44 adults with a history of NSSI, but no NSSI in the past year; 94% aged under 30 years; Female = 98%	N = 110 university students with no history of NSSI; 100% aged under 30 years; Female = 86%	Self-Injurious Thoughts and Behavior Interview (SITBI)	Experience of Shame Scale (ESS)
Todd (2002), UK	Cross-sectional	N = 73, male prisoners; Mean age; 30.88 years, SD = 7.08	–	Study specific measure of NSSI	Test of Self-Conscious Affect for Socially Deviant populations (TOSCA-SD); Other As Shamer scale (OAS)
VanDerhei et al. (2014), US	Cross-sectional	N = 378 university students; Mean age = 20.84 years, SD = 4.7; Female = 71%	–	Inventory of Statements About Self-Injury (ISAS)	Test of Self-Conscious Affect - 3 (TOSCA-3)
Weingarden, Renshaw, Wilhelm, Tangney, and DiMauro (2016), US	Cross-sectional	N = 114 adults with Body Dysmorphic Disorder; Mean age = 30.22 years, SD = 10.86; Female = 92% N = 114 adults with Obsessive Compulsive Disorder; Mean age = 30.60 years, SD = 10.66; Female = 86%	N = 133 health adult controls; Mean age = 36.44 years, SD = 13.28; Female = 76%	Suicide Behaviours Questionnaire - Revised (SBQ-R)	Test of Self-Conscious Affect - 4 (TOSCA-4)
Wiklander et al. (2012), Sweden	Cross-sectional	N = 337 psychiatric outpatients N = 108 patients with a BPD diagnosis and history of attempted suicide; Mean age = 29.9 years, SD = 8.0; Female = 97% N = 67 patients without a BPD diagnosis and history of attempted suicide; Mean age = 34.8 years, SD = 12.4; Female = 67% N = 162 patients without a history of attempted suicide; Mean age = 45.3 years, SD = 8.9; Female = 72%	N = 161 healthy controls; Mean age = 44.6 years, SD = 7.7; Female = 63%	Montgomery Åsberg Depression Rating Scale (MADRS-S)	Test of Self-Conscious Affect (TOSCA)
Xavier, Pinto Gouveia, and Cunha (2016), Portugal	Cross-sectional	N = 782 Middle and secondary school children; Mean age = 14.9 years; SD = 1.8 Female = 52.8%	–	Risk Taking and Self Harm Inventory for Adolescents - Portuguese Version (RTSHIA)	Other As Shamer scale – 2, Portuguese version (OAS-2)

BDI (Beck, Ward, Mendelson, Mock, & Erbaugh, 1961) = Beck Depression Inventory; DSHI (Gratz, 2001) = Deliberate Self-Harm Inventory; HIDS (Ross & Heath, 2002) = How I Deal with Stress Questionnaire; HQ (Resnick & Weaver, 1994) = Habit Questionnaire for self-injurious behaviour; IBS (Rossotto, Yager, & Rorty, 1994) = Impulsive Behavior Scale; ISAS (Klonsky & Glenn, 2009) = Inventory of Statements About Self-injury; MADRS-S (Svanborg & Asberg, 2001) = Montgomery-Asberg Depression Rating Scale; MSI-BPD (Zanarini et al., 2003) = Mclean Screening Instrument for BPD; PAI (Morey, 1991) = Personality Assessment Inventory; RTSHIA (Portuguese version: Xavier, Cunha, Pinto-Gouveia, & Paiva, 2013) = The Risk-Taking and Self-Harm Inventory for Adolescents Portuguese Version; SASII (Linehan, Comtois, Brown, Heard, & Wagner, 2006) = Suicide Attempt Self-Injury Interview; SBQ-R (Osman et al., 2001) = Suicidal Behavior Questionnaire Revised; SHI (Sansone & Sansone, 2010) = Self-harm Inventory; SIBQ (Johns & Holden, 1997) = Self-Injurious Behaviour Questionnaire; SIQ (Reynolds, 1987) = Suicide Ideation Questionnaire; SITB (Nock, Holmberg, Photos, & Michel, 2007) = Self-Injurious Thoughts and Behavior Interview. ESS (Andrews et al., 2002) = Experiences of Shame Scale; ISS (Cook, 1994) = Internalised Shame Scale; NEO-PI-R (Costa & McCrae, 1985) = the Revised NEO Personality Inventory; OAS (Goss, Gilbert, & Allan, 1994) = The Other as Shamer Scale; OAS-2 (Matos, Pinto-Gouveia, Gilbert, Duarte, & Figueiredo, 2015) = The Other as Shamer Scale-2 Portugese Version; OBCS (McKinley & Hyde, 1996) = Objectified Body Consciousness Scale; OBCS-Y (Lindberg, Hyde, & McKinley, 2006) = Objectified Body Consciousness Scale – Youth; PFQ-2 (Harder & Zalma, 1990) = the Harder Personal Feelings Questionnaire; SVQ (Brown et al., unpublished) = Shame Variability Questionnaire; TOSCA (Tangney et al., 1989) = Test of Self-Conscious Affect; TOSCA-3 (Tangney, Dearing, Wagner, & Gramzow, 2000) = Test of Self-Conscious Affect 3; TOSCA-SD (Hanson & Tangney, 1996) = Test of Self-Conscious Affect Socially Deviant; TOSCA-4 (Tangney et al., 2008) = Test of Self-Conscious Affect 4.

**Table 2:** Risk of bias assessment.

Authors	Unbiased cohort selection	Selection minimizes baseline differences in demographic factors	Sample size calculated	Validated method for ascertaining clinical status or participant group	Validated methods for assessing shame/guilt	Validated methods for assessing self-harm	Blind outcome assessment	Adequate follow-up period	Adequate handling of missing data	Appropriate analytic methods
Brown et al. (2009)	Unclear	N/A	No	Yes	Yes	Yes	No	Yes	No	Yes
Bryan, Morrow, et al. (2013)	Yes	N/A	No	Yes	Yes	Yes	Yes	N/A	Yes	Yes
Donhauser (2007)	Partial	N/A	Yes	N/A	Yes	Yes	N/A	N/A	Yes	Yes
Duggan et al. (2015)	Yes	Yes	No	Yes	Yes	Partial	No	Yes	Yes	Yes
Erchull et al. (2013)	No	N/A	No	N/A	Yes	Yes	Yes	N/A	Yes	No
Fieldman (1989)	Yes	N/A	No	Yes	Partial	Yes	No	N/A	Unclear	Yes
Gandy (2013)	Partial	N/A	Yes	Yes	Yes	Yes	Yes	N/A	Yes	Yes
Etzel (2004)	Partial	N/A	No	No	Yes	Yes	No	N/A	Unclear	Yes
Izadi (2014)	Partial	N/A	No	Yes	Yes	Yes	No	N/A	Yes	Yes
Kealy et al. (unpub.)	Yes	N/A	No	Yes	Yes	No	No	N/A	Yes	Yes
Kealy et al. (2017)	No	N/A	No	Yes	Yes	Yes	No	N/A	Yes	Yes
Lamb (2004)	No	N/A	Yes	Yes	Yes	Yes	No	N/A	Yes	Yes
Lester (1998)	No	N/A	No	N/A	Yes	Unclear	No	N/A	Unclear	Unclear
Mallindine (2002)	Unclear	Yes	No	Yes	Yes	No	No	N/A	Yes	Yes
McLeod (2002)	No	N/A	No	N/A	Yes	Yes	Partial	N/A	Yes	Yes
Milligan and Andrews (2005)	No	N/A	No	Yes	Yes	Partial	No	N/A	Yes	Yes
Nelson & Muehlenkamp et al. (2012)	No	N/A	No	N/A	Yes	Yes	Yes	N/A	Yes	Yes
Paulsen (2013)	Partial	N/A	No	Yes	Yes	Partial	Yes	N/A	Yes	Yes
Pritchard (2009)	No	N/A	No	Yes	Yes	Yes	Yes	N/A	Yes	Yes
Rusch et al. (2007)	Unclear	N/A	No	Yes	Yes	No	No	N/A	Yes	Yes
Rutherford (2015)	Partial	N/A	Yes	N/A	Yes	No	Yes	N/A	Yes	Yes
Schaefer (2014)	Yes	N/A	No	Yes	Yes	Partial	Partial	N/A	Unclear	Yes
Schoenleber (2013)	Partial	N/A	No	Yes	Yes	Yes	No	N/A	Yes	Yes
Seidlitz et al. (2001)	Yes	N/A	No	Yes	Yes	No	No	N/A	Unclear	Yes
Taylor, Jomar, et al. (2018)	No	N/A	No	Yes	Yes	Yes	No	N/A	Yes	Yes
Todd (2002)	Partial	Yes	No	Yes	Yes	No	No	N/A	Yes	Yes
VanDerhei et al. (2014)	No	N/A	No	N/A	Yes	Yes	No	N/A	Yes	Yes
Weingarden et al. (2016)	No	N/A	No	Yes	Yes	No	Yes	N/A	Yes	Yes
Wiklander et al. (2012)	Unclear	No	No	Yes	Yes	No	No	NA	Yes	Yes
Xavier et al. (2016)	Unclear	N/A	No	N/A	Yes	Partial	Yes	N/A	Yes	Partial

N/A = Not Applicable.

A broad variety of measures capturing distinct subtypes of shame and guilt were assessed across the included studies. The most common was shame or guilt proneness, typically assessed using the TOSCA (Tangney, Wagner, & Gramzow, 1989) or later versions of this measure (*k* = 11). Body-related shame was the second most commonly assessed subtype of shame, usually measured using the Experiences of Shame Scale (k = 4; ESS; Andrews et al., 2002). A number of studies also assessed exposure to general feelings of shame or guilt (not linked to a particular aspect of the self) using the Personal Feelings Questionnaire (k = 6; PFQ2; Harder & Zalma, 1990). Within this review we refer to this as ‘state shame’ and ‘state guilt’, to distinguish it from other forms. This broad range of emotion subtypes meant that few studies were identified focusing on any one subtype, and thus limited the number of studies contributing to any one meta-analysis.

### 3.2 Risk of bias

The assessment of risk of bias is presented in Table 2. Overall, risk of bias was relatively low with regards to the data that were the focus of this review. Notably, for unpublished data sets the information was not always available to ascertain the risk of bias associated with these data. The most common methodological problems related to justification of sample size, the use of heavily self-selecting samples (e.g. participants responding to flyers or online advertisements), blinding of researchers, and measurement of self-harm. Only four studies justified their sample size in terms of power calculations. This may mean that analyses were underpowered in some cases, leading to inflated Type II error rates. Attempts at blinding researchers or interviewers to participants' status were rarely undertaken, which may have introduced rater bias and expectancy effects. Although most studies still employed widely used and validated tools to assess self-harm status, around a third used single-item (sometimes unvalidated) self-report measures, which may have led to misclassification.

In terms of methodological strengths, three of the four studies involving group comparisons attempted to match groups on key socio-demographic variables (e.g. age, gender, ethnicity, socio-economic status). Hence confounding variables are unlikely to have biased group comparisons. Furthermore, all but one study used a valid method for ascertaining the clinical status or participant group, and most studies used valid and reliable measures to rate shame and/or guilt. Missing data also appeared minimal (i.e. < 20%) for a large proportion of studies, and in cases where missing data was apparent, appropriate details were provided in terms of how this was managed (e.g. use of imputation strategies to minimize bias). Finally, the analytic techniques adopted were appropriate in the large majority of studies.

### 3.3 Association between shame, guilt and NSSI

Table 3 reports the bivariate association between shame, guilt, and self-harm, grouped in terms of emotion subtype and outcome. Where data on two or more comparable associations were identified, a random-effects meta-analysis was undertaken to produce aggregate effect sizes. Bivariate effects for individual studies are reported in Supplementary Table 1. When meta-analyses were repeated using REML rather than DL estimation results were very similar (see Supplementary Table 2).

**Table 3:** Summary of bivariate associations between Shame or Guilt variables and self-harm.

Shame or guilt variable	Outcome	*N*/*K*	Association	*I*^*2*^
Shame proneness	NSSI frequency	488/4	***r* = 0.25 (95% CI: 0.08, 0.40)**	48%
	NSSI history (binary)	493/3	***d* = 0.42 (95% CI: 0.24, 0.60)**	0%
	Suicide attempt history (binary)	1306/4	***d* = 0.36 (95% CI: 0.05, 0.66)**	80%
Body shame	NSSI frequency	239/2	*r* = 0.07 (95% CI:. -30, 0.42)	85%
	NSSI history (binary)^⁎^	826/5	*d* = 1.61 (95% CI: −0.32, 3.55)	99%
	Suicide attempt frequency	119/1	***r* = 0.27 (95% CI: 0.10, 0.43)**	NA
	Self-harm history (binary)	89/1	***d* = 1.24 (95% CI: 0.78, 1.70)**	NA
External shame	NSSI frequency	782/1	***r* = 0.39 (95% CI: 0.33, 0.45)**	NA
	NSSI history (binary)	105/2	***d* = 0.51 (95% CI: 0.12, 0.90)**	0%
Characterological or internal shame	NSSI frequency	62/1	***r* = 0.33 (95% CI: 0.09, 0.54)**	NA
	NSSI history (binary)	205/1	***d* = 1.71 (95% CI: 1.39, 2.03)**	NA
	Suicide attempt frequency	119/1	***r* = 0.27 (95% CI: 0.10, 0.43)**	NA
	Self-harm history (binary)	119/2	***d* = 0.39 (95% CI: 0.02, 0.77)**	0%
	Self-harm frequency	20/1	*r* = −0.38 (95% CI: −0.70, 0.08)	NA
State shame	Suicide attempt history (binary)	278/3	*d* = 0.58 (95% CI: −0.12, 1.27)	74%
	Self-harm history (binary)	137/1	***d* = 0.37 (95% CI: 0.03, 0.70)**	NA
Performance, appearance and relationship related shame	Suicide attempt frequency	236/1	***r* = 0.20–0.26 (95% CI: 0.07, 0.38)**	NA
Guilt proneness	NSSI frequency	386/2	*r* = −0.01 (95% CI: −0.11, 0.09)	0%
	NSSI history (binary)	360/1	*r* = −0.07 (95% CI: −0.17, 0.03)	NA
	Suicide attempt history (binary)	1335/5	*d* = 0.12 (95% CI: −0.08, 0.32)	51%
State guilt	Suicide attempt history (binary)	363/4	***d* = 0.59 (95% CI: 0.25, 0.93)**	33%
	Self-harm history (binary)	137/1	***d* = 0.47 (95% CI: 0.13, 0.81)**	NA

Note: NSSI = non-suicidal self-injury; *K* refers to independent samples rather than studies; Meta-analysis undertaken where two or more studies available. Effects in bold are significant at *p* < .05; * Included one study with unusually large effect size. Exclusion of this study result in *d* = 0.35 (−0.10, 0.79), *I*^*2*^ = 81%.

Individuals with a history of NSSI reported greater levels of shame proneness, characterological shame, and external shame, with moderate to large effect sizes. No significant difference was apparent for body-related shame (*k* = 5 studies). This lack of difference was largely informed by the study by Duggan and colleagues in school students (2015), whose data suggests little overall difference in body shame at time 2 between those with and without a history of NSSI. However, Duggan, Heath, and Hu (2015) did report significant longitudinal effects, discussed below. There was also an unusually large effect size for a single study looking at body shame, linked to unusually small standard deviations (Nelson & Muehlenkamp, 2012). One study focused on external shame in a high secure inpatient sample noted that self-reported external shame was lower than is typically seen in the general population, though it is unclear whether this reflects a response bias or a characteristic of the population (Mallindine, 2002). When all studies investigating the relationship between shame (irrespective of subtype) and NSSI history were included together in a meta-analysis (*k* = 10), a large association was identified, *d* = 1.09 (0.17, 2.01), *I*^*2*^ = 98%, but this reduced to a more moderate effect when the one study with unusually small standard deviations (Nelson & Muehlenkamp, 2012) was excluded, *d* = 0.47 (0.17, 0.78), *I*^*2*^ = 82%, and with REML estimation, *d* = 0.48 (0.20, 0.76).

There were also small to moderate positive associations between NSSI frequency and shame proneness, external shame and characterological shame. However, again, there was no significant relationship with body shame based on a meta-analysis of two studies (Etzel, 2004; Pritchard, 2009). When all studies investigating the relationship between shame (irrespective of subtype) and NSSI frequency were included together in a meta-analysis (*k* = 7), a moderate association was identified, *r* = 0.24 (0.06, 0.40), *I*^*2*^ = 88%, and with REML estimation, *r* = 0.24 (0.07, 0.39). In summary, there is evidence that shame (with the possible exception of body shame) is elevated in those with a history of NSSI, and to a lesser extent, associated with the frequency of NSSI. This was apparent across a range of populations including university and high school students, survivors of sexual abuse and domestic violence, inpatients, adults with experiences of NSSI, and people in prison. However, most studies relied on small samples (*k* = 7 with *n* 〈100) and many shame subtypes were investigated by only one or two studies, making these findings preliminary.

In contrast to shame, only two studies of undergraduate students examined the relationship between guilt and NSSI. Guilt proneness was not related to either NSSI history or frequency (Schoenleber, 2013; VanDerhei, Rojahn, Stuewig, & McKnight, 2014).

### 3.4 Association between shame, guilt and suicidal behaviour

Shame proneness was elevated in participants with a history of suicide attempts compared to those without, but not state shame (see Table 3). These two meta-analyses demonstrated high inconsistency, suggesting that effect sizes are moderated by other factors. The number of studies contributing to these meta-analyses were too low to warrant statistical testing of moderating variables (Higgins & Green, 2009), but it is notable that smaller effects were apparent for one study focused on women with a borderline personality disorder (BPD) diagnosis (Rusch et al., 2007). It is possible that because experiences of shame are already elevated in those diagnosed with BPD (Rizvi, Brown, Bohus, & Linehan, 2011) the subsequent association with suicidal behaviour is attenuated. Two studies also found associations between shame and suicide attempt frequency, reporting small but significant positive correlations with shame related to character, body, performance, appearance and relationships (McLeod, 2002; Rutherford, 2015). When all studies investigating the relationship between shame (irrespective of subtype) and suicidal behaviour were included together in a meta-analysis (*k* = 8), a moderate association was identified, *d* = 0.49 (0.27, 0.70), *I*^*2*^ = 70%, and with REML estimation, *d* = 0.48 (0.28, 0.69). Overall, there is evidence of a small relationship between some forms of shame and suicidal behaviour, but this evidence is limited, and further confirmation is required.

State guilt, but not guilt proneness, was also elevated in those with a history of suicide attempts compared to those without (See Table 3). When all studies investigating the relationship between guilt (irrespective of subtype) and suicidal behaviour were included together in a meta-analysis (*k* = 8), a small association was identified, *d* = 0.29 (0.06, 0.51), *I*^*2*^ = 67%, and with REML estimation, *d* = 0.32 (0.06, 0.60).

### 3.5 Association between shame, guilt and self-harm

A subset of three studies measured self-harm, rather than NSSI or suicidal behaviour more specifically (Kealy, 2019; Lamb, 2004; Milligan & Andrews, 2005). Two out of three studies reported significantly greater levels of shame in those with a history of self-harm than those without (see Table 3). Shame was not significantly correlated with self-harm frequency in one study of psychiatric outpatients (Lamb, 2004), and the direction of the trend was actually negative (greater guilt related to less frequent self-harm), though the sample was very small (*n* = 20) increasing the risk of unusual and unrepresentative results. Unpublished data also indicated elevated state guilt in psychiatric outpatients with a history of self-harm (Kealy, 2019).

### 3.6 Adjusted associations

The association between shame and NSSI (frequency or history) remained significant even after adjusting for guilt in most analyses (5 out of 6). In a sample of undergraduate students, appearance-related shame emerged as the only significant correlate of suicide attempt history (OR = 1.04) when adjusting for shame and guilt proneness, relationships, and performance related shame (McLeod, 2002). However, another study of individuals with a history of suicidal thoughts found no significant relationship between shame proneness and suicidal behaviour when adjusting for guilt proneness (*r* = 0.20; Izadi, 2014). It is unclear if the form of correlation used in this study is suitable for a binary outcome like suicide attempt history (i.e. point-biserial or tetrachoric). Across two studies, shame remained significantly positively associated with NSSI whilst adjusting for negative affect (alongside guilt-proness; OR = 2.12; Schoenleber, 2013) or internalizing symptoms (alongside guilt proneness and demographic information; OR = 1.37, 95% CI: 1.05–1.78; VanDerhei et al., 2014). A single study found that both guilt and shame were not correlated with suicidal behaviour whilst adjusting for depressive symptoms (Lester, 1998). As before, it was not clear if appropriate correlation coefficients were used given the binary outcome.

Guilt proneness did not have a significant bivariate association with NSSI history. This association remained non-significant when adjusting for shame proneness in one study (Schoenleber, 2013), and a negative association with NSSI history emerged in another study (OR = 0.76, 95% CI: 0.62–0.94), whereby greater guilt proneness was related to a lower risk of NSSI when adjusting for shame proneness (VanDerhei et al., 2014). Guilt proneness was also not associated with NSSI when adjusting for negative affect (alongside shame proneness; OR = 0.69, *p* = .17; Schoenleber, 2013).

### 3.7 Longitudinal associations

Only two studies adopted longitudinal methodologies. Duggan et al. (2015) investigated body shame over one year in high school students. They found that shame did not distinguish between those with an NSSI history who had not maintained this behaviour compared to controls with no history of NSSI. In contrast, shame remained elevated in those who continued to engage in NSSI relative to the control participants. Brown and colleagues (Brown, Linehan, Comtois, Murray, & Chapman, 2009) followed up a small sample of women diagnosed with BPD. Higher state shame, assessed using items from the PFQ-2, was associated with almost twice the risk of subsequent NSSI (relative risk ratio = 1.88, 95% CI: 1.04–3.38) within a survival analysis. This association did not remain significant when adjusting for feelings of fear. Non-verbal indicators of shame (rated based on video recorded interviews) were associated with NSSI occurrence in non-adjusted analyses (relative risk ratio = 1.99, 95% CI: 1.07–3.69), and remained significant when fear and sadness were also adjusted for (relative risk ratio = 1.86, *p* < .05).

## 4 Discussion

The aim of the current study was to provide a systematic review and meta-analysis of the available literature regarding self-harm and its relationships with shame and guilt. Thirty papers were identified for inclusion. Individuals with a history of NSSI typically reported greater shame across a range of different populations and shame sub-types, compared to those without a history of NSSI. Body shame was an exception, where evidence of an association was less clear and varied more dramatically between studies. Shame was also positively correlated with frequency of NSSI engagement. Effect sizes were typically small to moderate according to Cohen's rules of thumb (Cohen, 1988). These associations between shame and NSSI typically held whilst adjusting for co-occurring feelings of guilt and mood-related symptoms. There was also evidence that shame had a positive association with both suicidal behaviour and self-harm (where measured as a general construct), but studies were fewer and results more varied. In contrast, results were mixed regarding the association between guilt and self-harm (including NSSI and suicidal behaviour). Guilt proneness did not appear to be associated with NSSI or suicidal behaviour, but state guilt was associated with suicide attempt history across four studies. Whilst shame appears linked to self-harm, the lack of longitudinal studies limits conclusions about the direction or temporal characteristics of these associations. The two longitudinal studies identified suggested that feelings of shame may contribute to the risk of NSSI over time.

The results are largely consistent with wider research, where shame has been found to be positively associated with a wide range of mental health difficulties, whilst results regarding guilt have been more ambiguous (Blythin et al., 2018; Carden et al., 2018; Kim et al., 2011; Pugh et al., 2015). This review did exclude some more specific forms of guilt, however, including trauma-related guilt, which may have more of a pronounced relationship with mental health difficulties (Pugh et al., 2015). The decision to exclude these experiences was taken in light of their distinct phenomenology, and the potential confounding effects of event-specific factors. For example, there is evidence the type of trauma can moderate the relationship between guilt and suicidal thinking (Bryan, Ray-Sannerud, Morrow, & Etienne, 2013). It was therefore anticipated that the inclusion of such studies would blur the relationships between key variables and impact upon the generalisability of findings. Overall, guilt has received less research attention than shame, and so we would recommend researchers study shame and guilt in tandem where possible to further establish any differential associations with mental health difficulties.

The results indicate that elevated experiences of shame are associated with self-harm behaviour. As the data are observational and correlational it is not possible to conclude that feelings of shame actively drive or maintain self-harm. These data are consistent with emotion-regulation orientated models of self-harm, which view self-harm as a potential response to aversive affective states like shame (Chapman et al., 2006; Hasking et al., 2017; Nock, 2009), and with people's self-reported reasons for self-harm, that most commonly concern managing negative internal states including shame (Breen, Lewis, & Sutherland, 2013; Curtis, 2016; Taylor, Jomar, et al., 2018). Nonetheless, experiences of shame could also be a consequence of self-harm (e.g. self-injury scar-related shame; Bachtelle & Pepper, 2015), or an epiphenomenon related to other processes that drive self-harm. Qualitative research highlights how shame may both be an antecedent and consequence of self-harm (Curtis, 2016). It is also important to recognise that the functions of self-harm vary widely (Taylor, Jomar, et al., 2018) and that different emotions may play a greater or lesser role for different people. Theory suggests that shame may be particularly relevant because it is inherently aversive and closely tied to how individuals perceive and relate to themselves. Self-harm may emerge as a means of regulating these self-directed feelings. There is evidence that greater endorsement of shame-regulation reasons for NSSI (i.e. reducing shame) is associated with greater NSSI frequency (Schoenleber, 2013; Schoenleber & Berenbaum, 2012). These results are also consistent with evidence that a more hostile or critical style of relating to oneself is a risk factor for some forms of self-harm (Forrester, Slater, Jomar, Mitzman, & Taylor, 2017).

In summary, this review highlights that shame and self-harm are linked, but caution should be taken in making further conclusions at this stage. Future research may benefit from moving beyond cross-sectional designs to better understand this relationship. Whilst causality cannot be ascertained within observational data, further evidence of temporality (i.e. that shifts in shame or guilt preceded subsequent changes in self-harm behaviour over time) would support a stronger case for a potentially causative relationship. Research methodologies that enable a more fine-grained investigation of how individuals respond to experiences of shame in the moment, such as experience sampling methodologies, could also be beneficial (Pratt & Taylor, 2019).

The review identified a number of methodological issues that were apparent in the extant literature. These include the variety of measures used to assess key constructs, namely shame. It is unclear the extent to which many of these measures tap meaningfully distinct constructs. Due to this uncertainty we did not plan to combine different types of guilt or shame into common meta-analyses. This heterogeneity of measures limits the extent to which comparisons between studies can be made and is problematic for summarising effects. It is suggested that researchers within this field would benefit from adopting a common set of measures across studies. This would facilitate comparisons regarding the severity and impact of guilt and shame across studies and population groups.

A further issue relates to the cultural sensitivity of the shame measures utilised within the reviewed studies. Cultural differences in both the precipitants to and manifestations of shame have been highlighted, underscoring the potential role of cultural expectations in the experience of shame (Abu-Kaf & Priel, 2008; Brown, 2006). However, most measures used within reviewed literature were both developed for and tested within predominantly western, individualist cultures. As a result, the extent to which these measures can be considered both sensitive and generalizable to a range of cultures is limited.

The use of meta-analysis provided a means of summarising the size and strength of the association between shame or guilt and self-harm. Whilst this approach has advantages over simple “vote-counting” of significant effects (taking into account the size of studies and the degree of heterogeneity), limitations should also be noted. The meta-analyses, especially those focused on emotion subtypes (i.e. body-related shame, external shame, etc.) included small numbers of studies, and as such lack precision. Moreover, in many cases high inconsistency was present, but there were not enough studies available to examine possible moderators that may explain this. We also conducted larger meta-analyses by grouping emotion subtypes together, but it should be noted that these may also obscure important differences between subtypes (e.g. body-related shame had a weaker association with NSSI). Overall, these results should therefore be interpreted with caution. Whilst they arguably provide a starting estimate of the size of the bivariate association between these constructs, additional studies would help further confirm these associations. Larger-scale replications of earlier studies adopting the same measures and populations would be particularly beneficial given the current diversity in both populations and measures used.

This review is also limited by the exclusion of non-English language research. The current review also focussed solely on quantitative research. A review of the qualitative research concerning shame and self-harm may shed further light on the potential mechanisms underlying this relationship. Many studies used correlation coefficients to capture the relationship with self-harm frequency. For this reason, the correlation coefficient was typically used as the metric within our meta-analyses. However, in many cases self-harm frequency may be better represented as a count variable, with a Poisson or negative binominal distribution. In such cases correlation coefficients may not capture associations as well, introducing more error. Whilst it was beyond the scope of this review to focus on suicidal ideation as well as behaviour, there may be benefits to also reviewing this literature, particularly where studies examine the transition from thoughts to behaviour.

If shame does contribute to the onset and maintenance of self-harm, then approaches to self-harm intervention and prevention that focus on feelings of shame may be effective. In terms of direct intervention, compassion-focused therapy (CFT) has been developed specifically for individuals who struggle with shame and self-criticism (Gilbert, 2009). There is preliminary evidence that individuals undergoing CFT experience reductions in shame (Judge, Cleghorn, McEwan, & Gilbert, 2012; Leaviss & Uttley, 2015). Other therapeutic approaches that focus on tolerance of difficult emotional states, such as Dialectical Behaviour Therapy (Rizvi et al., 2011), or focus on self-directed feelings and relating, such as Cognitive Analytic Therapy (Sheard et al., 2000), may also be helpful. Given the heterogeneity in the triggers, functions and forms of self-harm (Klonsky & Muehlenkamp, 2007; Taylor, McDonald, Smith, Nicholson, & Forrester, 2019), a personalised approach, adopting shame-focused interventions where this appears to be part of the mechanism underlying that individual's self-harm, is likely to be better than a one-size-fits-all approach. At a societal level, recognition of and support for groups where experiences of shame and self-harm are elevated, such as those in the Lesbian, Gay, Bisexual or Transgender (LGBT) community may be beneficial (McDermott, Roen, & Scourfield, 2008; Taylor, Dhingra, Dickson, & McDermott, 2018a). Campaigns and programmes designed to reduce the stigma and shame may help in these instances.

This review summarises the extant literature concerning shame, guilt and self-harm. We provide a preliminary indication of the direction and magnitude of the association between these emotions (and their subtypes) and self-harm. We also highlight key gaps in the literature and future directions, including the need for longitudinal designs and larger-scale replications of earlier studies adopting the same measures and populations.

## Role of funding source

Funding supporting this study was provided by Medical Research Council (*MR/N006062/1*)*. The* MRC had no role in the study design, collection, analysis or interpretation of the data, writing the manuscript, or the decision to submit the paper for publication.

## Contributors

Dr. Sheehy was involved in conceptualisation, design and planning of the review (including protocol development), literature searches and screening, data extraction, synthesis and evaluation, and write-up. Dr. Dhingra, Ms. Noureen and Ms. Khaliq were involved in screening, data extraction, quality checking, evaluation and write-up. Dr. Pontin and Prof. Husain were involved in supervision, planning, and write-up. Dr. Cawley was involved in conceptualisation, planning and evaluation. Dr. Taylor was involved in project management, supervision, conceptualisation, design and planning of the review, literature searches and screening, data extraction, synthesis and evaluation, and write-up.

## Declaration of Competing Interest

None.

## Appendix A Supplementary data

Supplementary material Image 1
