# Supplementary material for: An examination of the relationship between shame, guilt and self-harm: A systematic review and meta-analysis
Source: Clin Psychol Rev. 2019 Nov;73:101779. doi: 10.1016/j.cpr.2019.101779 (PMC6891258; doi:10.1016/j.cpr.2019.101779)
Supplement: Supplementary file 1 — Supplementary material [file mmc1.docx]

Supplementary Table 1

Summary of Bivariate Associations between Shame or Guilt Variables and Self-Harm by Individual Study

| Study | Shame or guilt variable (measure) | Outcome | Standardized association | Non-standardized association |
| --- | --- | --- | --- | --- |
| Bryan et al (2013) | State shame (PFQ2)  State Guilt (PFQ2) | Suicide attempt history | *d* = 1.25*  *d* = 1.15* | Mean difference = 8.90  Mean difference = 5.93 |
| Duggan et al (2015)^a^ | Body shame (OBCS-Y) | NSSI history | Males: *d* = -0.03  Females: *d* = 0.01 | Males: Mean difference = -0.04  Females: Mean difference = 0.01 |
| Erchull et al (2013) | Body shame (OBCS) | NSSI history | *r* = .18* |  |
| Fieldman  (1989) | Guilt proneness (Guilt Scale) | Suicide attempt history | *d* = 1.13* | Mean difference = 18.3 |
| Gandy  (2014/2013) | Shame proneness (TOSCA-3) | NSSI frequency  NSSI history | *r* = .26*  *r* = .25* |  |
| Etzel  (2004) | Characterological shame (ESS)  Body shame (ESS) | NSSI frequency | *r* = .33*  *r* = .27* |  |
| Kealy et al  (unpub.) | State shame (PFQ2)  State guilt (PFQ2) | Self-harm history  Self-harm history | *d* = 0.37*  *d* = 0.47* | Mean difference = 2.71  Mean difference = 2.19 |
| Kealey et al (2017) | State shame (PFQ2)  State guilt (PFQ2) | Suicide attempt history  Suicide attempt history | *d* = 0.51  *d* = 0.52 | Mean difference = 3.20  Mean difference = 2.29 |
| Lamb  (2004) | Internal shame (ISS) | Self-harm frequency  Self-harm history | *r* = -.38  *d* = 0.09 | -  Mean difference = 1.03 |
| Mallindine  (2002) | External shame (OAS) | NSSI history | *d* = 0.53 | Mean difference = 8.40 |
| McLeod (2003) | Performance, appearance and relationship related shame | Suicide attempt frequency | *r* = .20-.26* |  |
| Milligan & Andrews  (2005) | Characterological shame (ESS)  Body shame (ESS) | Self-harm history  Self-harm history | *d* = 0.49*  *d* = 1.24* | Mean difference = 3.94  Mean difference = 4.35 |
| Nelson & Muehlenkamp  (2012)^b^ | Body shame (OBCS) | NSSI history | *d* = 6.86* | Mean difference = 0.59 |
| Paulson  (2013) | Shame proneness (TOSCA-3) | NSSI frequency | *r* = .15 |  |
| Pritchard (2014) | Body shame (OBCS) | NSSI frequency | *r* = -.11 |  |
| Rusch et al (2007) | Shame proneness (TOSCA-3)  Guilt proneness (TOSCA-3)  State shame (PFQ2)  State guilt (PFQ2) | Suicide attempt history  Suicide attempt history  Suicide attempt history  Suicide attempt history | *d* = -0.26  *d* = -0.24  *d* = -0.04  *d* = 0.52 | Mean difference = -1.76  Mean difference = -1.07  Mean difference = -0.24  Mean difference = 1.93 |
| Rutherford  (2016) | Characterological shame (ESS)  Body shame (ESS) | Suicide attempt frequency  Suicide attempt frequency | *r* = .27*  *r* = .27* |  |
| Schaefer  (2014) | Shame proneness (TOSCA-SD)  Guilt proneness (TOSCA-SD) | Suicide attempt frequency  Suicide attempt frequency | *r* = .09  *r* = .02 |  |
| Schoenleber  (2013) | Shame proneness (TOSCA-3)  Guilt proneness (TOSCA-3) | NSSI frequency  NSSI history  NSSI frequency | *r* = .58*  *d* = .53*  *r* = 0.14 | -  Mean difference = 0.30 |
| Seidlitz et al  (2001) | Guilt proneness (NEO-PI-R) | Suicide attempt history | *d* = 0.34 | Mean difference = 1.08 |
| Taylor et al (2018) | Characterological shame (ESS)  Body shame (ESS) | NSSI history  NSSI history | *d* = 1.71*  *d* = 0.89* | Mean difference = 13.01  Mean difference = 3.24 |
| Todd  (2002) | External shame (OAS) | NSSI history | *d* = 0.50* | Mean difference = 7.21 |
| VanDerhei et al (2014) | Shame proneness (TOSCA-3)  Shame proneness (TOSCA-3)  Guilt proneness (TOSCA-3)  Guilt Proneness( TOSCA-3) | NSSI frequency  NSSI history  NSSI frequency  NSSI history | *r* = .16*  *r* = .19*  *r =* -.02  *r* = -.07^c^ |  |
| Weingarden et al (2016) | Shame proneness (TOSCA-4)  Guilt proneness (TOSCA-4) | Suicide attempt history  Suicide attempt history | *d* = 0.61*  *d* = 0.07 | Mean difference = 19.14  Mean difference = 1.31 |
| Wiklander et al (2012) | Shame proneness (TOSCA)  Guilt proneness (TOSCA) | Suicide attempt history  Suicide attempt history | *d* = 0.60*  *d* = 0.16 | Mean difference = 5.93  Mean difference = 1.13 |
| Xavier et al (2016) | External shame (OAS-2) | NSSI frequency | *r* = .39* |  |

Note: NSSI = non-suicidal self-injury; ESS = Experience of Shame Scale; ISS = Internalised Shame Scale; NEO-PI-R = NEO Personality Inventory Revised; OAS = Other As Shamer Scale; OAS-2 = The Other as Shamer Scale-2 Portugese Version ; OBCS = Objectified Body Consciousness Scale; OBCS-Y = Objectified Body Consciousness Scale – Youth; TOSCA = Test of Self-Conscious Affect; TOSCA-SD = Test of Self-Conscious Affect for Socially Deviant populations; * *p* < .05; ^a^ Means for those with a history of NSSI are based on aggregating data from individuals who either continued NSSI or stopped NSSI at follow-up; ^b^ standardized effect size should be treated with caution due to unusually small reported standard deviations; ^c^ value reported as significant in paper but on inspection coefficient not large enough to be significant given sample size (assuming two-tailed test and α = .05).

Supplementary Table 2

Summary of Bivariate Associations between Shame or Guilt Variables and Self-Harm with REML Estimation

| Shame or guilt variable | Outcome | *N*/*K* | Association | *I^2^* |
| --- | --- | --- | --- | --- |
| Shame proneness | NSSI frequency | 488/4 | ***r* = .24 (95% CI: .09, .38)** | 41% |
|  | NSSI history (binary) | 493/3 | ***d* = 0.42 (95% CI: 0.24, 0.60)** | 0% |
|  | Suicide attempt history (binary) | 1306/4 | ***d* = 0.36 (95% CI: 0.04, 0.68)** | 82% |
| Body shame | NSSI frequency | 239/2 | *r* = .07 (95% CI: .-30, .42) | 85% |
|  | NSSI history (binary) | 826/5 | *d* = 1.61 (95% CI: -.97, 4.20) | >99% |
| External shame | NSSI history (binary) | 105/2 | ***d* = 0.51 (95% CI: 0.12, 0.90)** | 0% |
| Characterological or internal shame | Self-harm history (binary) | 119/2 | ***d* = 0.39 (95% CI: 0.02, 0.77)** | 0% |
| State shame | Suicide attempt history (binary) | 278/3 | *d* = 0.58 (95% CI: -0.13, 1.29) | 75% |
| Guilt proneness | NSSI frequency | 386/2 | *r* = -.01 (95% CI: -.11, .09) | 0% |
|  | Suicide attempt history (binary) | 1335/5 | *d* = 0.10 (95% CI: -0.02, 0.21) | 0% |
| State guilt | Suicide attempt history (binary) | 363/4 | ***d* = 0.59 (95% CI: 0.25, 0.93)** | 33% |

Note: NSSI = non-suicidal self-injury; *K* refers to independent samples rather than studies; Meta-analysis undertaken where two or more studies available. Effects in bold are significant at *p* < .05;
